# Supplementary material for: Comprehensive profiling of the gut microbiota in patients with chronic obstructive pulmonary disease of varying severity
Source: PLoS One. 2021 Apr 9;16(4):e0249944. doi: 10.1371/journal.pone.0249944 (PMC8034725; doi:10.1371/journal.pone.0249944)
Supplement: S1 Fig — (DOCX) [file pone.0249944.s001.docx]

**S1 Fig 1.** OTU Venn diagram


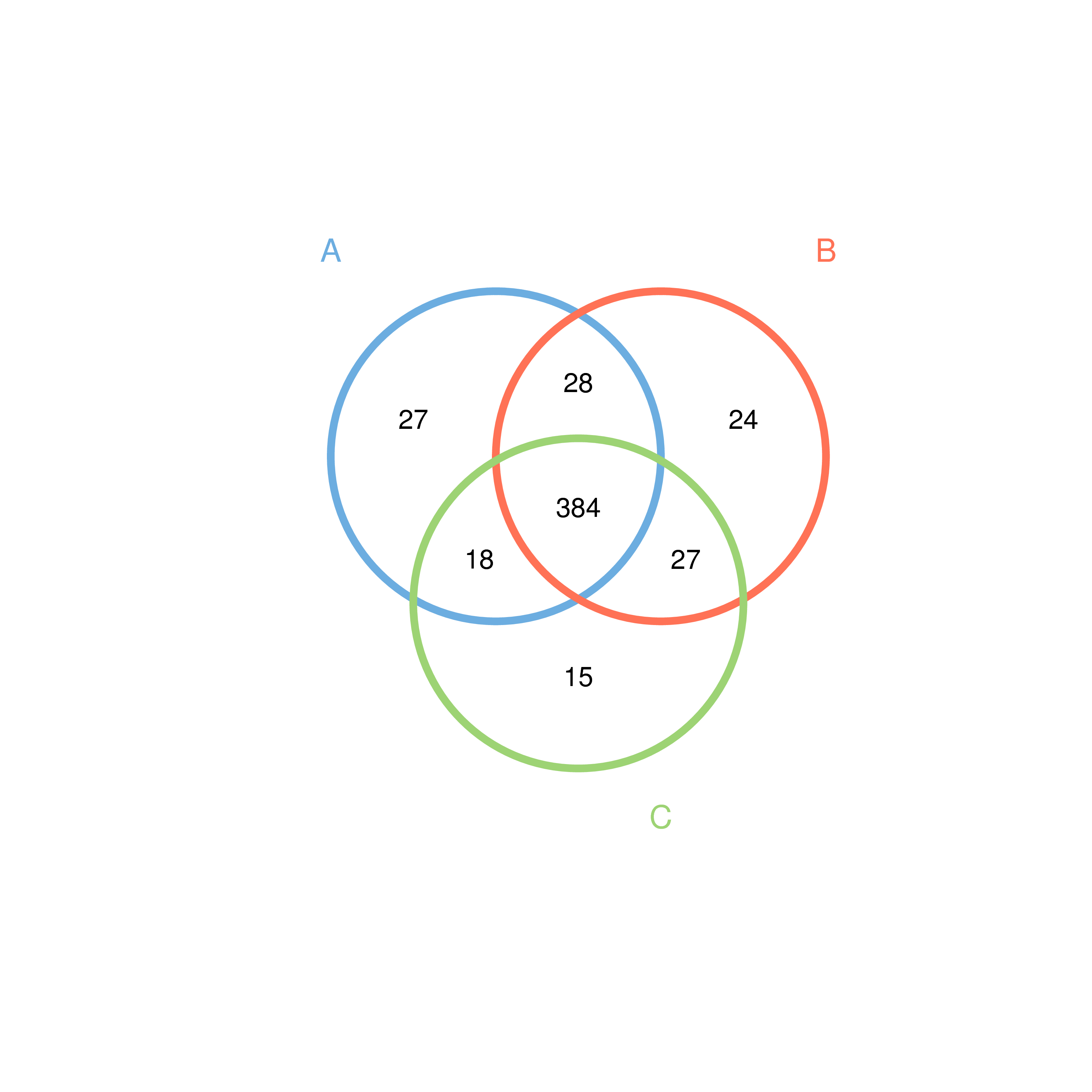


A: stage 1, B: stage 2, C: stage 3+4; The numbers represent the number of OTUs unique or common to each sample or group.
