# Supplementary material for: Comprehensive profiling of the gut microbiota in patients with chronic obstructive pulmonary disease of varying severity
Source: PLoS One. 2021 Apr 9;16(4):e0249944. doi: 10.1371/journal.pone.0249944 (PMC8034725; doi:10.1371/journal.pone.0249944)
Supplement: Attachment — Submitted filename: Response to comments R2.docx [file pone.0249944.s005.docx]

Reviewer #2: Major

Apologies, but I don’t see where the raw data has been deposited (e.g. NCBI sequence read archive). I think this is a PLoS requirement.

**Response**: In this study, we reported the analyses of 16S rRNA V3+V4 hypervariable regions by sequencing for stool from COPD patients. The data is neither microarray nor deep sequencing data. We did not found any public database which enrolled microbiota profile using partial sequence (V3+V4) data.

Data Availability: The data has been also submitted as S1 Table (Excel file) in Supporting Information files. The data file contains the reads number blast to the OTUs in each sample. The sample ID: A- x = samples in group A; B- x = samples in group B; C- x = samples in group C

Some articles had been published in Plos One (as following) did not mention any deposited (e.g. NCBI sequence read archive) record.

- Ingrid S. Surono, Dian Widiyanti, Pratiwi D. Kusumo, Koen Venema. Gut microbiota profile of Indonesian stunted children and children with normal nutritional status. Published: January 26, 2021https://doi.org/10.1371/journal.pone.0245399
- Aasia Khaliq, Resmi Ravindran, Samia Afzal, Prasant Kumar Jena, Muhammad Waheed Akhtar, Atiqa Ambreen, Yu-Jui Yvonne Wan, Kauser Abdulla Malik, Muhammad Irfan, Imran H. Khan Gut microbiome dysbiosis and correlation with blood biomarkers in active-tuberculosis in endemic setting. Research Article | published 22 Jan 2021 PLOS ONE https://doi.org/10.1371/journal.pone.0245534

We are willing to share our data by fasta format under a reasonable requirement in the future.

Minor

The authors state that “The R version 3.1.1 was used for heatmaps”.

That’s not really sufficient as one needs to know what package was used. The authors, at minimum, should specify what R packages were used in their analysis (e.g. pheatmap). A succinct summary (listing all R packages) in the methods would suffice.

**Response:**

Thank you for the suggestion. The statement has been revised as “The pheatmap package (https://cran.r-project.org/src/contrib/Archive/pheatmap/) was used for ecological analysis and heatmaps”. (Page 7)

Re: Reply 7:

If not already included somewhere, the authors should state the average DNA concentration observed in sample DNA extracts. If it is quite high (50ng/ul or more) this serves as a counterargument to the influence of contamination, which could also be highlighted in the discussion. While it is now field standard to control for contamination, and the exclusion of such samples is a major oversight, it would be comforting to know your DNA yields from stool were high, which presumably they were.

**Response**:

We added the DNA extraction kit in methods section (page 4). The average DNA concentration has been stated in results section as “The stool DNA samples were eluted in 200 μl AE buffer. The average of DNA concentration was 5.52 ng/μl (range 1.05 – 12.43 ng/μl).” Please see page 8.
