# Supplementary material for: Comprehensive profiling of the gut microbiota in patients with chronic obstructive pulmonary disease of varying severity
Source: PLoS One. 2021 Apr 9;16(4):e0249944. doi: 10.1371/journal.pone.0249944 (PMC8034725; doi:10.1371/journal.pone.0249944)
Supplement: Attachment — Submitted filename: Response to comments R3.docx [file pone.0249944.s006.docx]

Reviewer #2:

I cannot see any reason that public repositories such as the sequence read archives (NCBI) would not accept the type of data you describe. I highly encourage the authors to make their data publicly available as per field standards.

ACADEMIC EDITOR: The manuscript is almost ready for acceptance but the comment made by reviewer 2 needs to be addressed. It is now field standard for all 16S rRNA sequencing data to be uploaded onto a public repository server (eg European Nucleotide Archive) so that other researchers have open access to the data. Please organise for your data to be submitted and available in this way.

Response:

We have organized our data and submitted to European Nucleotide Archive, Accession No: PRJEB43280. Please see the section “Availability of data and materials”.
